# Atypical memory B cell frequency correlates with antibody breadth and function in malaria immune adults

**DOI:** 10.1038/s41598-024-55206-2

**Published:** 2024-02-28

**Authors:** Frederica Dedo Partey, Jasmine Naa Norkor Dowuona, Abigail Naa Adjorkor Pobee, Melanie Rose Walker, Belinda Aculley, Diana Ahu Prah, Michael Fokuo Ofori, Lea Klingenberg Barfod

**Affiliations:** 1 Noguchi Memorial Institute for Medical Research, University of Ghana, Legon, Ghana; 2 Centre for Medical Parasitology, Department of Immunology and Microbiology, Faculty of Health and Medical Sciences, University of Copenhagen, Copenhagen, Denmark; 3 West African Centre for Cell Biology of Infectious Pathogens, University of Ghana, Legon, Ghana

**Keywords:** Atypical memory B cells, Malaria, Antibody breadth, Malaria antibody avidity, Diseases, Infectious diseases, Malaria, Infectious diseases, Malaria

## Abstract

Clinical immunity to malaria develops slowly after repeated episodes of infection and antibodies are essential in naturally acquired immunity against malaria. However, chronic exposure to malaria has been linked to perturbation in B-cell homeostasis with the accumulation of atypical memory B cells. It is unclear how perturbations in B cell subsets influence antibody breadth, avidity, and function in individuals naturally exposed to malaria. We show that individuals living in high malaria transmission regions in Ghana have higher *Plasmodium falciparum* merozoite antigen-specific antibodies and an increased antibody breadth score but lower antibody avidities relative to low transmission regions. The frequency of circulating atypical memory B cells is positively associated with an individual's antibody breadth. In vitro growth inhibition is independent of the ability to bind to free merozoites but associated with the breadth of antibody reactivity in an individual. Taken together, our data shows that repeated malaria episodes hamper the development of high avid antibodies which is compensated for by an increase in antibody breadth. Our results provide evidence to reinforce the idea that in regions with high malaria prevalence, repeated malaria infections lead to the broadening of antibody diversity and the continued presence of atypical memory B cell populations.

## Introduction

Humoral responses that lead to the production of antibodies are critical in protective immunity against the erythrocytic stage of *Plasmodium falciparum* infections^1–3^. It has been demonstrated in passive transfer studies where the administration of antibodies from semi-immune adults to symptomatic children resulted in the resolution of febrile illness and subsequent decrease in parasite density^4^. Several immuno-epidemiological studies have therefore sought to delineate the characteristics of humoral responses in naturally acquired immunity to malaria. Identifying targets of the protective antibodies and understanding the mechanism of antibody function is further critical for the development of malaria vaccines, that induce better protection than the current RTS, S vaccine^5–7^. Merozoite antigens are key targets of naturally acquired protective immunity making them promising candidates for vaccine development. Moreover, increasing antibody breadth (the number of antigens to which an individual has higher antibody titres) is linked to protection against malaria^7,8^. Also, studies have shown that the breadth of antibody reactivity correlates with transmission intensity implying that repeated exposure increases one’s breadth of antibody reactivity^7,9^.

Antibody avidity, a measure of the cumulative binding strength between the antibody and antigen is derived from the affinities of numerous individual non-covalent interactions^10^. Also, antibody avidity reflects the degree of affinity maturation of antibodies in the germinal centers and impacts malarial immunity^11^. Generally with the classical understanding of B cell development, recurrent exposure to an antigen should lead to germinal center reactions driving several rounds of affinity maturation and thereby increasing antibody avidity^12^. However, evidence suggests that chronic exposure to *P. falciparum* infections which interferes with B cell function may disrupt germinal center reactions and compromise affinity maturation^13–15^. Research has shown that repeated exposure to *P. falciparum* infection is linked to alterations in B cell sub-set homeostasis which includes the proliferation of atypical MBCs^15,16^. Atypical memory B cells are a subclass of B cells that lack the surface markers CD21 and CD27. Alterations in the expression of CD11c, CXCR3, CXCR5, inhibitory receptors, and transcription factors such as Tbet are another characteristic of atypical MBC^14,17,18^. Atypical B cells are generally thought to be dysfunctional with a diminished ability to secrete antibodies in vitro upon stimulation^19^. It is however not known how alterations in B cell subsets influence antibody breadth and avidity of malaria antibodies.

Drawing on our current knowledge of humoral immunity in malaria, we propose that affinity maturation may be impeded in areas of high transmission. However, this limitation may be offset by an increase in antibody breadth. In the present study, we examined how malaria transmission intensity alters peripheral B cell subsets and the relationship with breadth of antibody reactivity, avidities of merozoite-antigen, and antibody function in partially immune adults.

## Results

### Demographic and parasitological characteristics of study participants.

The study was a crossectional study conducted in two communities in Ghana; Accra and Cape Coast. Accra is a region of low malaria transmission intensity, with an entomological inoculation rate (EIR) less than 50 infective bites per person per year^20^. Although the exact EIR for Cape Coast is not known, the Ghana Malaria Indicator Survey conducted in 2019 revealed an 18% malaria prevalence in children living in Cape Coast, compared to a mere 2% prevalence in Accra^21^. Volunteers were enrolled in the study during two seasons; October–November, 2020 (minor rainy season) and May–July 2021(major rainy season). A total of 200 healthy asymptomatic adults were recruited for the study. The median age of the study subjects was 29 years (Table 1). The malaria prevalence among the study population tested by RDT was 4% of which all 8 individuals were from Cape Coast. Estimation of parasitaemia using the more sensitive photo-induced electron polymerase chain reaction (PET-PCR) shows 10% parasite prevalence among participants of which 9.5% were from Cape Coast with 0.5% from Accra. The mean haemoglobin concentration among the study participants was 13.5 g/dL (SD ± 1.9) (Table 1).

**Table 1:** Demographic and Clinical characteristics of study participants.

Variable	Accra	Cape coast	Total	*p*-value
N	73	127	200	
Median age _*_	26 (23–34)	30 (24–35)	29 (23–35)	0.1348
**Gender**				
Male n (%)	53 (72.6)	64 (50.4)	117 (58.5)	
Female n (%)	20 (27.3)	63 (49.6)	83 (41.5)	
**Parasitaemia**				
RDT Positive n (%)	0	8 (6.3)	8 (4)	
RDT Negative n (%)	73 (100)	119 (92.1)	192 (96)	
PCR Positive n (%)	1 (1.4)	19 (14.96)	20 (10)	
**Sickling**				
Positive n (%)	6 (8.2)	12 (9.4)	18 (9)	
Negative n (%)	67 (91.8)	115 (90.6)	182 (91)	
Haemoglobin g/dL (SD)_α_	13.321(1.3836)	13.55 (2.11)	13.483 (1.9245)	0.9146

### Merozoite antigen-specific antibody levels in plasma and seroprevalence in two areas of varying malaria transmission intensity

**Figure 1:**
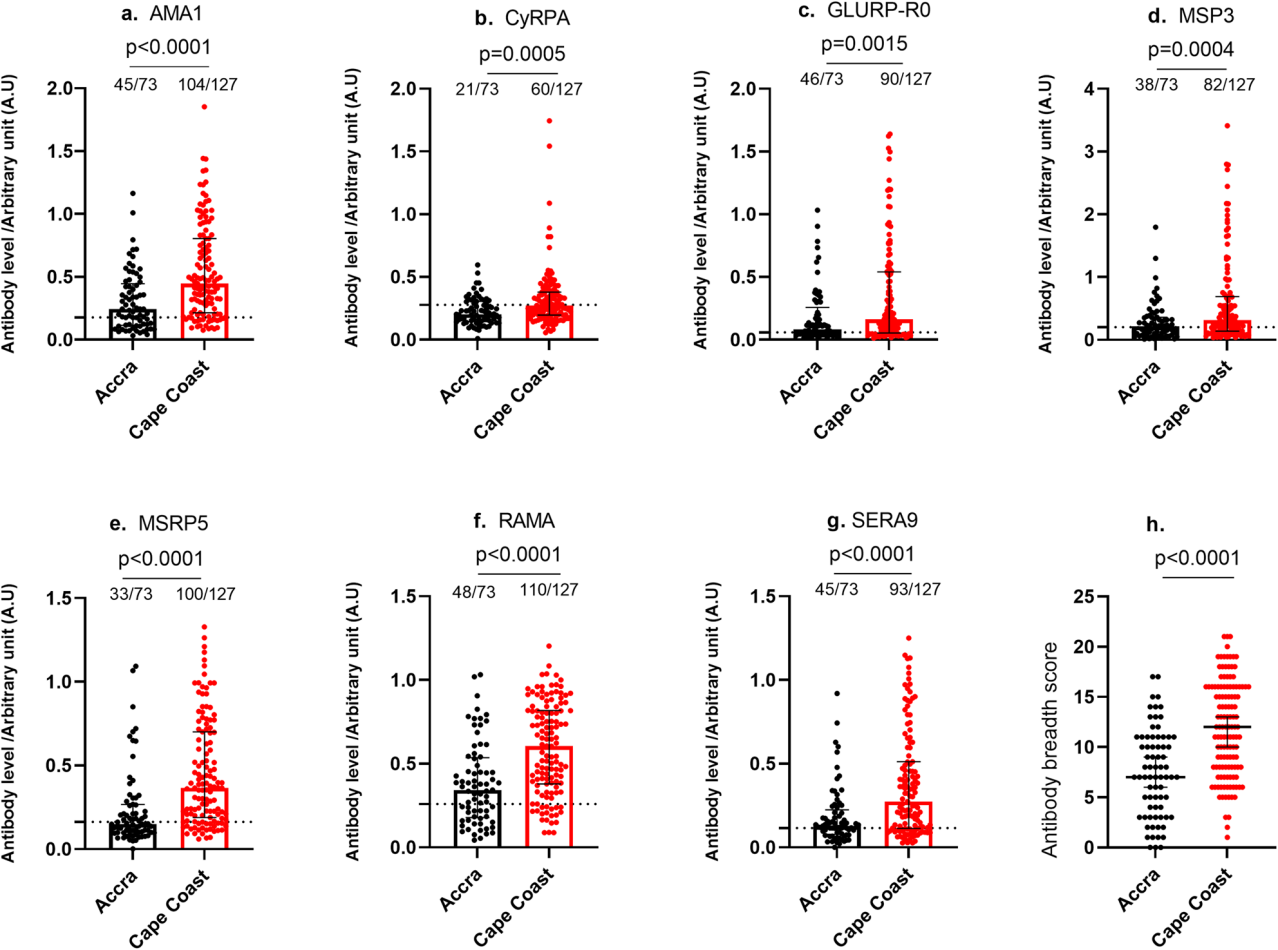
Plasma IgG reactivity to specific antigens in two regions of different transmission intensities. *P. falciparum* merozoite antigens; AMA1, GLURP-RO, CyRPA, MSP3, MSRP5, SERA9, and RAMA IgG levels (Arbitrary Unit (A.U)) in malaria immune adults. Statistically significant differences between groups were represented by asterisks (*p* < 0.05(*), *p* < 0.01 (**), and *p* < 0.0001(****). The box plot shows the median IgG value and the data distribution using the quartiles. Bars represent the median IgG values represented in arbitrary units. The box represents the interquartile range showing the lower (1st) quartile and the upper quartile (3rd). The bars above the whiskers represent the extreme minimum and extreme maximum values with a 95% Confidence interval. Dashed line indicates cut-off for seropositivity based on mean OD + 2 × standard deviation of non-exposed controls.

Antibody responses to a panel of *P. falciparum* merozoite antigens, including AMA-1, MSP3, MSRP5, CyRPA, GLURP-RO, RAMA, and SERA9 were measured in participant plasma by ELISA (Fig. 1a–g). For all the antigens examined, the median plasma IgG antibody levels against the merozoite antigens were significantly higher among individuals living in the high transmission region compared to those from the low transmission region. Similarly, the prevalence of individuals who were seropositive for the antigens examined was mostly higher in the high transmission area than the low transmission area. In both Accra and Cape Coast, seroprevalence of AMA-1, MSP3, MSRP5, RAMA, and GLURP-RO were high in contrast to low seroprevalence of CyRPA and SERA9 in both the high and low transmission areas. Immunity to malaria requires the maintenance of high antibody magnitude and antibody responses to multiple parasite antigens. We, therefore, estimated the antibody breadth score amongst the study participants. As expected, the breadth score was significantly higher amongst individuals residing in the Cape Coast than individuals in Accra (Fig. 1h).

### An increase in antibody breadth is associated with lower CyRPA and MSRP5 antibody avidities

**Figure 2:**
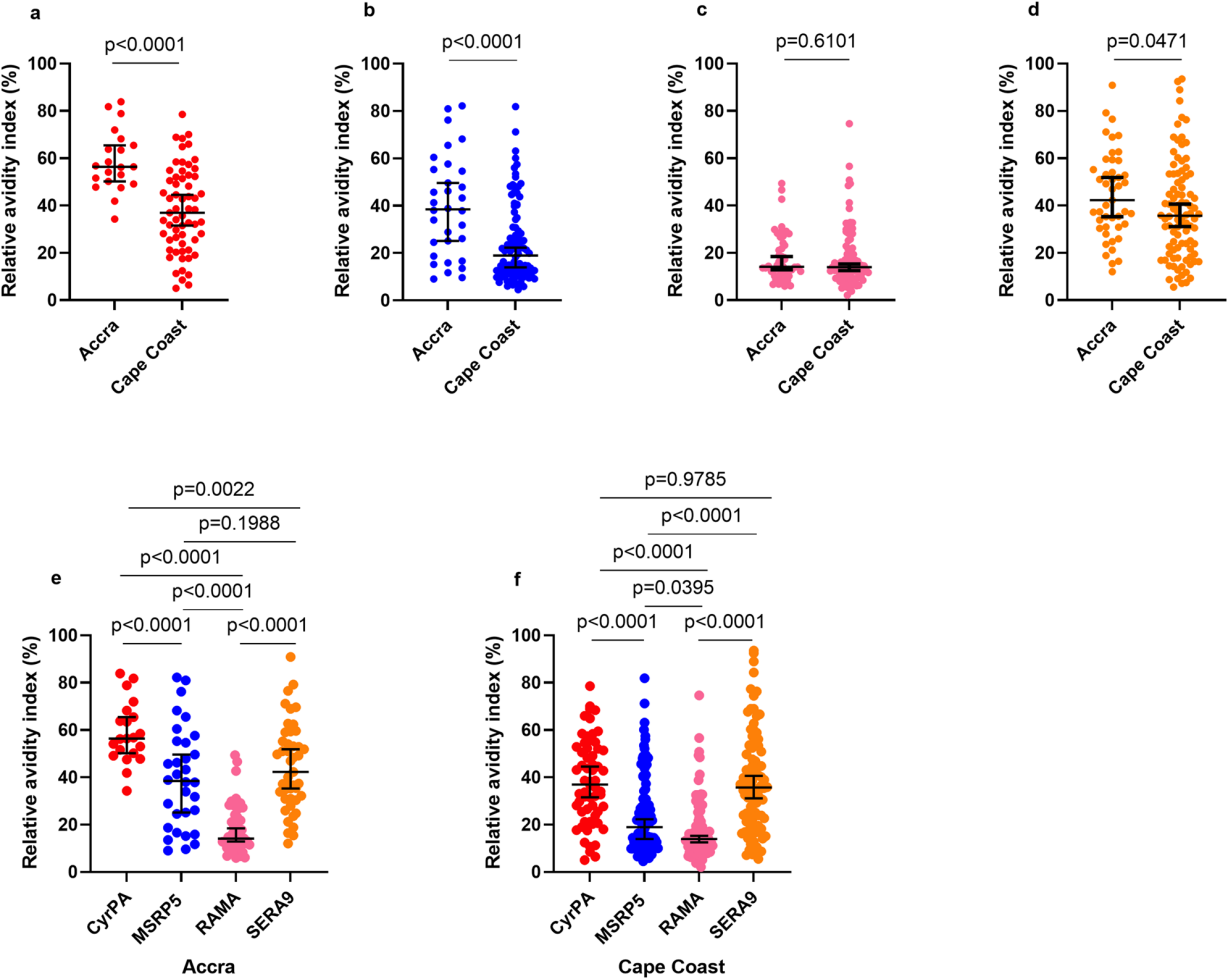
Individuals living in areas of low transmission develop high avid antibodies to merozoite antigens. Relative avidity index of antibodies between individuals residing in Accra and Cape Coast who are seropositive for CyRPA (**A**), MSRP5 (**B**), RAMA (**C**) and SERA9 (**D**). The Median Relative avidity index for all antigens is shown for Accra (**E**) and Cape Coast (**F**). Horizontal lines represent the median mark and error bars depict a 95% confidence interval. Bar and * indicate statistical significance.

**Figure 3:**
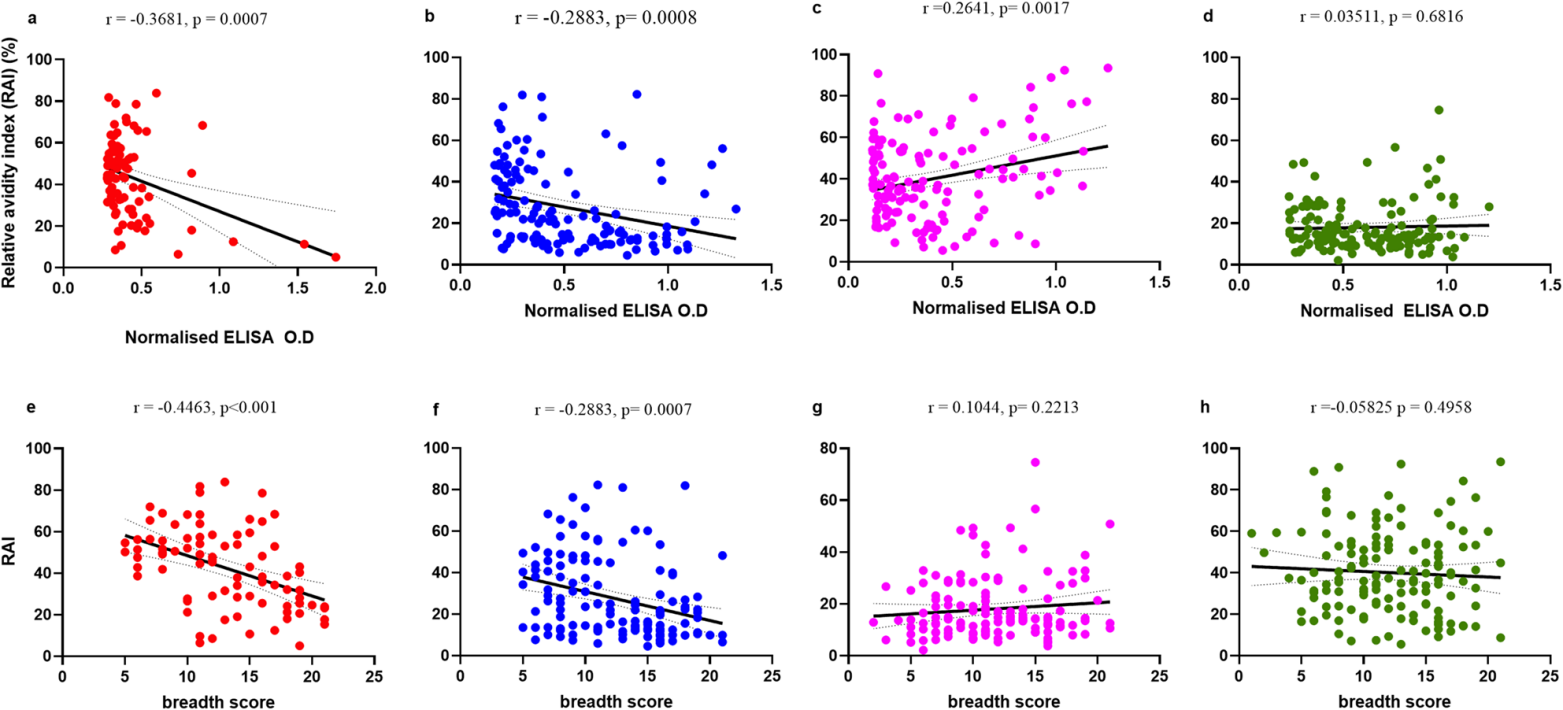
Relationship between relative avidity index and antibody breadth score. Linear regression analysis (solid line) and 95% confidence interval (dashed line) between antibody breadth score of individuals living in Accra (top panel) and Cape Coast (lower panel) and relative avidity index, RAI (%) specific for CyRPA (**A**, **E**), MSRP5 (**B**, **F**), RAMA (**C**, **G**) and SERA9 (**D**, **H**). Spearman’s correlation coefficient r and *p* values are shown for each plot.

We next measured the relative avidities of antibodies in individuals seropositive for CyRPA, RAMA, SERA9, and MSRP5 and examined the correlation with antibody magnitude (Fig. 2) and breadth score in both high and low malaria transmission areas (Fig. 3). We focused on these four selected antigens because several prior studies have examined the avidities of antibodies against the other antigens (AMA-1, MSP3)^22–24^. Contrary to the median IgG antibody levels, the median relative avidity index (RAI) was significantly higher for CyRPA (*p* < 0.0001), MSRP5 (*p* < 0.0001), and SERA9 (*p* = 0.0471) in Accra compared to Cape Coast. The median RAI in Accra for CyRPA, MSRP5, and SERA 9 were 56.38%, 38.48%, and 42.30% respectively. In Cape Coast, the median RAI was 36.96%, 18.95%, and 35.68% for CyRPA, MSRP5, and SERA9 respectively (Fig. 2a, b, d). There was no significant difference in the median RAI between high and low transmission areas for RAMA-specific IgG antibodies (Fig. 2c). In both transmission areas, the median RAI of antibodies against the poorly immunogenic antigens such as CyRPA and MSRP5 were significantly high relative to the highly immunogenic antigen RAMA (Fig. 2e,f). There was an inverse correlation between the RAI of CyRPA-specific antibodies, antibody magnitude (r = −0.3681, *p* = 0.0007) (Fig. 3a), and antibody breadth score (r = −0.4463, *p* < 0.001) (Fig. 3e). A similar pattern was observed for the correlation between RAI of MSRP5-specific antibodies and (r = −0.2883, *p* = 0.0008) (Fig. 3b) antibody levels and antibody breadth score (r = -0.2883, *p* = 0.0007) (Fig. 3f). We observed a positive correlation between RAI of RAMA-specific antibodies and antibody levels (r = 0.2641, *p* = 0.0017) (Fig. 3c) but no correlation between RAI and the individual antibody breadth score (Fig. 3g). No significant correlation was found between the SERA9 antibody magnitude and the RAI (r = 0.03511, *p* = 0.6816) (Fig. 3d) or the antibody breadth score (r = −0.05825, *p* = 0.4958).

### The frequency of circulating atypical B cell population correlates with the breadth of merozoite-antigen antibody responses

**Figure 4:**
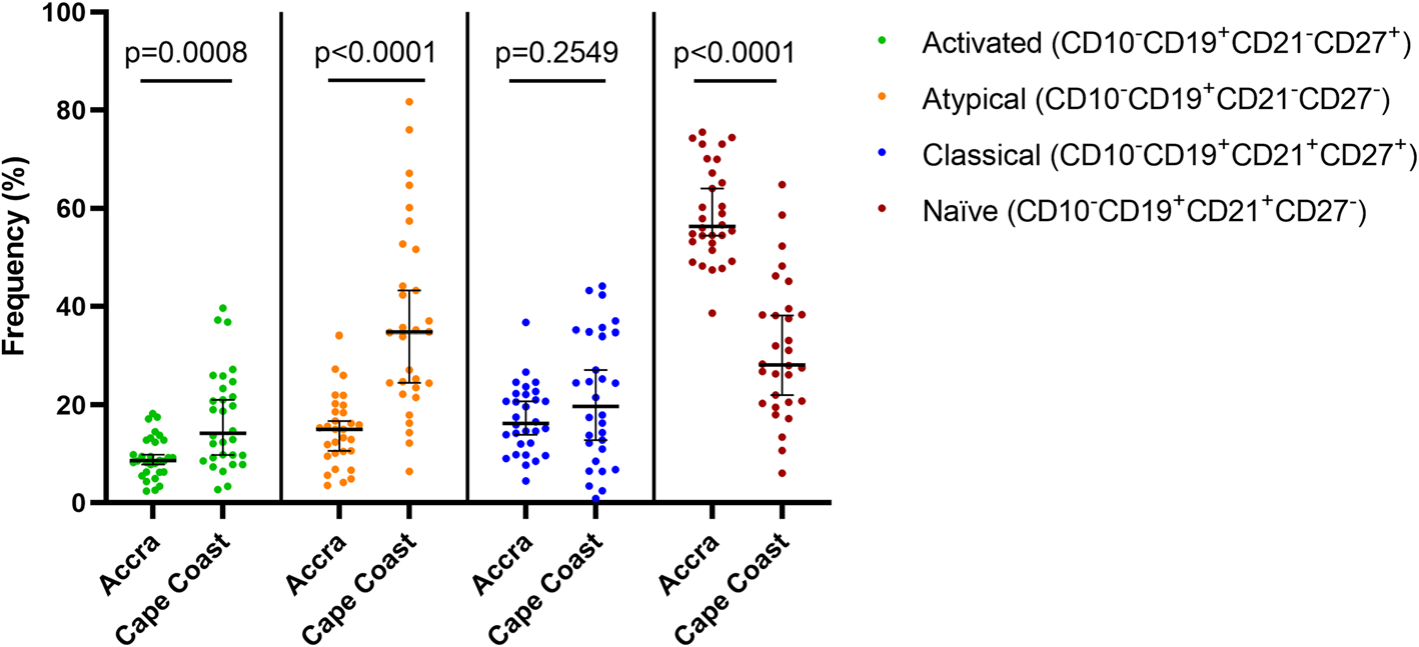
Frequency of peripheral B cell populations phenotypes. A scatter dot plot showing the Frequency of Atypical Memory B cells (CD27−, CD21−), Classical Memory B cells (CD21+, CD27+, Activated Memory B cells (CD21−, CD27+, and Naïve B cells (CD21+, CD27−) among the mature B cell population. Each individual is represented by a dot (n = 30 for each study site). The bars (colored) indicate the median frequency and the error bars (black) show the 95% confidence interval. Statistical significance was calculated using the Mann–Whitney U test. *P* values indicated on top of graph.

**Figure 5:**
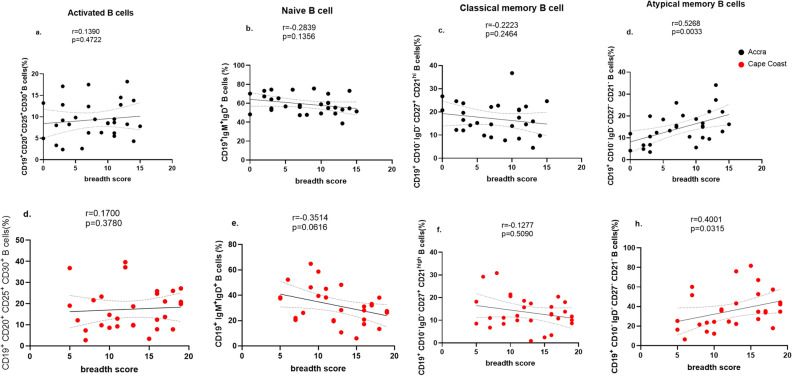
Correlations between the frequency of peripheral B cell populations and antibody breadth in both low and high malaria transmission areas. Linear regression analysis (solid line) and 95% confidence interval (dashed line) between antibody breadth score of individuals living in Accra (top panel) and Cape Coast (lower panel) and activated B cells (**A**, **D**), Naïve B cell (**B**, **E**), classical memory B cell (**C**, **F**) and atypical memory B cells (**D**, **H**). Spearman’s correlation coefficient r and *p* values are shown for each plot.

*P. falciparum* infection is known to disrupt the establishment of the B cell compartment resulting in the expansion of memory B cells with an atypical phenotype characterized by the expression of exhaustion markers^1,25^. We sought to examine the relationship between circulating B cell populations and the breadth of acquired antibodies amongst a subset of study participants for which there was adequate PBMCs living in high (N = 30) and low (N = 30) transmission areas (Fig. 4). There was a higher proportion of atypical memory B cells (*p*-value < 0.0001) and activated memory B cells (*p*-value = 0.0008) in the higher transmission intensity region as compared to those in the low transmission intensity region (Fig. 4). Naïve B cells (antigen inexperienced mature B cells) were significantly higher in individuals living in the low transmission region (*p* < 0.0001). However, there was no significant difference (*p* value = 0.2945) in the classical memory B cells population between the two sites. The increase in atypical memory B cells in individuals living in Cape Coast suggests that the expansion of atypical memory B cells is driven by continued parasite exposure. There was a positive correlation between the frequency of atypical memory B cell population and antibody breadth in both the low (r = 0.5268, *p* = 0.0033) and high transmission area (r = 0.4001, *p* = 0.0315) (Fig. 5d, h). The frequency of activated B cell, naïve B cell and classical memory B cell population did not correlate with the breadth of antibody responses in individuals in Accra (*p* = 0.4722, *p* = 0.1356, *p* = 0.2464)(Fig. 5a–c) and Cape Coast (*p* = 0.3780, *p* = 0.0616, *p* = 0.5090)(Fig. 5e,f,g) respectively.

### In vitro growth inhibition is independent of the ability to bind to free merozoites but associated with the breadth of antibody reactivity in an individual

**Figure 6:**
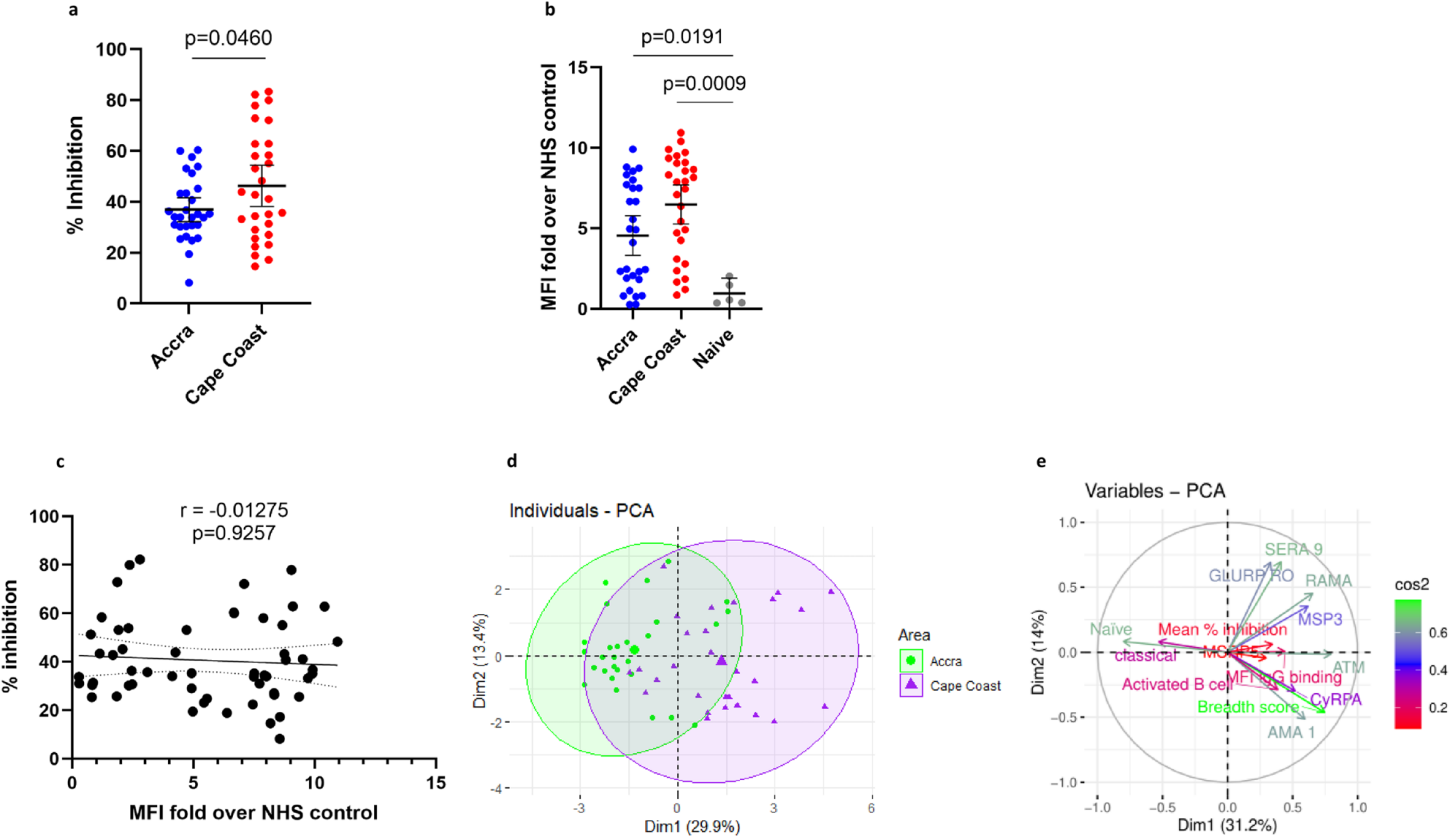
Relationship between malaria-specific antibody function and merozoite-antigen-specific antibody levels. Antibody function was assessed by the ability of immune plasma to inhibit in vitro parasite (3D7 strain) growth (**A**) and binding of free merozoites (**B**). Correlation between inhibition of in vitro parasite growth and binding of free merozoites among participants in Accra and Cape Coast (**C**). The first 2 principal component analyses were grouped by transmission intensity (Accra-green, Cape Coast-purple) (**D**). PCA analysis depicting the correlation between antigen-specific antibody levels, antibody function, and peripheral B cell population (**E**).

The polyfunctionality of malaria-specific antibodies was examined between the two transmission regions. First, we determined the abilities of plasma from malaria-exposed individuals to inhibit merozoite invasion in vitro (Fig. 6a). The median percentage inhibition in the high transmission area was 46.29% and significantly higher (*p* = 0.0460) than the median percentage inhibition in the low transmission area (36.93%). Secondly, we estimated the levels of total IgG in the plasma from the adults, that bind to the surface of free merozoites, by flow cytometry (Fig. 6b). The total binding IgG levels were higher among individuals living in high areas relative to the low transmission areas albeit not statistically significant in the present study (*p* = 0.0550) but were significantly higher for both areas compared to unexposed controls (*p* = 0.0493, *p* = 0.0013). We did not find a correlation between an individual’s ability to inhibit merozoite invasion in vitro and the total IgG binding (r = −0.01275, *p* = 0.9257, Fig. 6c).

Principal component analysis (PCA) of antibodies to explore the relationship between antibody function, merozoite antigen-specific antibody levels, and frequency of peripheral B cell populations did not discriminate individuals living in low transmission areas from the high transmission area (Fig. 6d). We thus, examined the correlations between antibody breadth score, antibody levels, frequency of peripheral B cell populations, and antibody function (Fig. 6e, Table 2.). There was a significant correlation between antibody breadth score (r = 0.3415, *p* = 0.0100), antibodies against AMA-1 (r = 0.3415, *p* = 0.0100), CyRPA, MSP3, and RAMA, and binding of IgG. The frequency of atypical memory- and naive B cells positively and negatively correlated with IgG binding respectively.

**Table 2:** Spearman rank correlation showing relationship between antigen-specific antibody levels and antibody function.

Antigen	Free merozoite binding			Growth inhibition assay		
	Spearman r	95% CI	*p*-value	Spearman r	95% CI	*p*-value
AMA-1	0.2684	−0.002078 to 0.5022	0.0455	0.1812	−0.09367 to 0.4304	0.1814
CyRPA	0.2988	0.03099 to 0.5266	0.0253	0.06494	−0.2090 to 0.3294	0.6344
GLURP−RO	0.07249	−0.2018 to 0.3362	0.5955	0.145	−0.1304 to 0.3996	0.2864
MSP3	0.3766	0.1184 to 0.5872	0.0042	0.1064	−0.1687 to 0.3662	0.435
MSRP5	0.09314	−0.1817 to 0.3545	0.4948	0.06022	−0.2135 to 0.3252	0.6593
SERA 9	0.1553	−0.1200 to 0.4085	0.2531	0.2276	−0.04554 to 0.4690	0.0917
RAMA	0.2705	0.0002068 to 0.5039	0.0438	0.2187	−0.05480 to 0.4617	0.1053

## Discussion

In the present study, we show that adults residing in areas of high malaria transmission intensity have significantly higher antibody levels and breadth of antibody reactivity to the studied merozoite antigens relative to adults living in areas of low malaria transmission intensity. The relative avidity of antibodies against CyRPA, MSRP5, RAMA, and SERA9 was higher in the low malaria transmission area than in the high transmission area. When we examined the peripheral B cell populations, atypical memory B cell populations were significantly expanded in individuals living in the high transmission areas and were found to strongly correlate with the breadth of antibody responses. The breadth of antibody reactivity, frequency of atypical memory B cells, and antibodies against AMA-1, RAMA, and CyRPA were associated with the ability of total IgG to bind free merozoites but not growth inhibitory activity.

The merozoite antigens examined presently are potential malaria vaccine candidates. AMA-1 MSP3, GLURP R0, and CyRPA have been extensively studied as either mono-antigen or multi-antigen vaccine candidates^26–28^ which are in different phases of clinical trials. SERA9, RAMA, and MSRP5 are emerging blood-stage vaccine candidates with potential synergistic abilities in combination vaccines. Recent studies by our group revealed that RAMA-specific monoclonal antibodies acted synergistically with RH5- and CyRPA-specific monoclonal antibodies in eliciting in vitro parasite neutralization^29^. We found SERA9, RAMA, and MSRP5 to be immunogenic in natural infections as both individuals living in high and low malaria transmission areas showed antibody reactivity to these merozoite antigens. The merozoite antigen-specific antibody seropositivity recorded in our study corresponds with data from previous studies in adults and children^5,30^.

It is well established that continuous exposure to *P. falciparum* infections drives the expansion of atypical memory B cells similar to non-classical memory B cells found in other chronic infections such as HIV^31^, hepatitis^32^and Tuberculosis^33^. Individuals naturally exposed to malaria infections were found to have higher proportions of circulating atypical memory B cells compared to malaria naïve individuals. Here, we found individuals from high malaria transmission areas having higher proportions of atypical memory B cells in line with earlier studies^34–37^. The function of atypical memory B cells in malaria immunity remains unclear. Some studies have shown atypical memory B cells to be dysfunctional with an inability to secrete antibodies in vitro^31^ and known to express inhibitory molecules such as Fc receptor-like 3 (FcRL3) and FcRL5^19^. In contrast, other studies have demonstrated that atypical memory B cells produce broadly neutralizing antibodies in vivo^34^ expanded in response to acute infections and correlates with antigen burden. Among our participants, there was a positive correlation between antibody breadth score and the frequency of atypical memory B cell population. These findings further support the notion that in areas of intense malaria, recurrent malaria drives an expansion of antibody repertoire and persistence of atypical memory B cell populations which do not contract before another episode of malaria happens. We did not estimate the frequency of antigen-specific memory B cells in this study, which is a limitation. Nonetheless, a previous study did not find a correlation between plasma antigen-specific IgG levels and antigen-specific memory B cells^37^.

Antibody affinity maturation is an essential characteristic of humoral immune response ensuring that reinfection results in the production of high-affinity antibodies. Relative avidity indexes of antibodies against the studied merozoite antigens except RAMA were significantly higher in Accra where malaria transmission is low compared to Cape Coast. Studies by others have recorded similar patterns to some merozoite antigens^22^ but found no difference in avidity to other merozoite antigens. This suggests intrinsic properties of an antigen could impact the avidity of the binding antibody. Previous studies in Ugandan children and adults living in high transmission areas found age-related effects on antibody avidity against some antigens (EBA-175 and MSP1-42) but not others (MSP2 and MSP3)^23^. Studies that have compared malaria antibody avidity between areas of differing transmission have largely focused on blood-stage antigens^22,23^. It would be interesting to know if the avidity of antibodies against antigens of pre-erythrocytic and sexual stage show a similar pattern as blood stage antigens.

Plasma from adults living in high malaria transmission areas significantly inhibited parasite growth and invasion in vitro relative individuals living in the low transmission areas. The ability of an individual’s plasma to inhibit parasite growth and invasion in the present study was not impacted by the individual’s antibody breadth or antibody levels against the panel of antigens studied. The findings from the present study are in line with previous studies that did not find any associations between growth inhibitory activity and antibodies against other merozoite antigens^6,38^. In contrast, some studies found a correlation between an individual’s antibody breadth and growth inhibitory activity^39^. In addition, antibodies against several other merozoite antigens including AMA-1^40,41^, erythrocyte binding ligand (EBL), and reticulocyte homolog (RH) family^39^ proteins have been associated with in vitro growth inhibitory activity in natural infections and vaccinations. A review of the current literature shows that the lack of correlation between growth inhibitory activity and antigen-specific antibody levels is not unusual considering the ELISA binding and functional assays used are evaluating different properties of naturally acquired immunity^38,42,43^. Conversely, antibody breadth, antibodies against AMA-1, CyRPA, and RAMA were associated with total IgG binding to free merozoites. This suggests that antibodies acquired during natural infections might be involved in antigen-specific functional mechanisms. It is interesting to note that antibodies against CyRPA, a poorly immunogenic antigen, are associated with antibody binding to free merozoites. Our findings further support the investigation of CyRPA as a blood-stage vaccine candidate.

In summary, we show that adults living in regions of high malaria transmission intensity have an increased antibody magnitude and breadth which is positively associated with the frequency of circulating atypical memory B cells. Low malaria transmission intensity drives high avidity antibodies against CyRPA, MSRP5, RAMA, and SERA9. The breadth of antibody reactivity, frequency of circulating atypical memory B cells, and antibodies against AMA-1, CyRPA, and RAMA were antibody features that correlated with total IgG binding of free merozoites.

## Materials and methods

### Study sites

This community-based cross-sectional study was carried out in two regions in Ghana; the Greater Accra Region and Cape Coast with varying *P. falciparum* transmission intensities. The Greater Accra region has a relatively low transmission intensity with a reported entomological inoculation rate (EIR) of < 50 infective bites/person/year^44^. Although the EIR of Cape Coast is unknown, the Ghana malaria indicator survey in 2019 recorded a malaria prevalence of 18% in children living within the Cape Coast compared to a 2% prevalence in Accra^45^. Thus, Cape Coast was designated as a high transmission relative to Accra in the study. In Cape Coast, blood samples were collected from individuals in two study sites—Efutu in the Cape Coast North District and Moree in the Abura Asebu—Kwamankese district. In Accra, participants were sampled from Legon and its environs in the La-Nkwantanang district of the Greater Accra Region.

### Study participants and sampling

Healthy adults aged between 18 and 60 years who had been residents in the study sites for at least 5 years and consented to participate in the study were enrolled. Pregnant women and participants with chronic diseases or those on any medication known to alter their immune system were excluded from the study. A total of 200 individuals were sampled from the two study sites comprising 127 from Cape Coast and 73 from the Greater Accra region. Of those from Cape Coast, thirty-seven (37) samples were collected from Efutu, and ninety (90) samples were collected from Moree. Participants were screened with the One Step Malaria HRP2/pLDH (P.f/Pan) kit (Wondfo, China). Venous blood was drawn into heparinized tubes. Heparinized blood was centrifuged at 720×*g* for 10 min to separate plasma and stored at −80 °C until ready to be used. Peripheral blood mononuclear cells (PBMC) were purified using the gradient centrifugation method on Ficoll Paque (GE Healthcare, Biosciences AB, Sweden). The PBMCs were cryopreserved in 90% fetal bovine serum (FBS) containing 10% dimethyl sulfoxide (DMSO) using controlled-gradient freezing equipment as previously described by^46^ and then transferred into liquid nitrogen tanks for long-term storage until ready to be used. Dried blood spots were also obtained for DNA extraction which was used for Polymerase Chain Reaction (PCR) for the determination of sub-microscopic infections. Interviews were conducted using a structured questionnaire to obtain demographic data and physical examinations including body temperature, height, and weight were also recorded. The study was approved by the Institutional Review Board of the Noguchi Memorial Institute for Medical Research (NMIMR-IRB CPN 079/18-19). Participation in the study was voluntary and informed consent was obtained from all volunteers before taking part in the study. All methods were carried out in accordance with relevant guidelines and regulations.

### *Plasmodium falciparum* merozoite antigens

Recombinant extracellular domains of merozoite antigens MSRP5-bio-his (Addgene plasmid #50805), SERA9-bio-his (Addgene plasmid #50820), RAMA-bio-his (Addgene plasmid #50737), and CyRPA-bio-his (*PF*D1130w-bio-his, Addgene plasmid #50823) were gifts from Dr. Gavin Wright (University of Oxford, Oxford, UK)^47^. Plasmids were transiently expressed using the Expi293 Expression System as described previously^48^. The expression of recombinant GLURP-RO (amino-terminal domain aa 24-489) AMA1, and MSP3 (3D7) have been extensively described elsewhere^49^.

### Antibody reactivity by Enzyme-linked Immunosorbent assay

Plasma antibody (IgG) levels specific for selected merozoite antigens were measured using a previously described ELISA assay^49^ with a few modifications. A panel of seven different *P. falciparum* merozoite antigens; AMA1, CyRPA, GLURP- RO, MSP3, MSRP5, RAMA and SERA9 were included in this study. In brief, 96-well NUNC Immuno maxisorp ELISA plates (Thermo Fisher Scientific, Denmark) were coated with 1 µg/ml antigen in Phosphate Buffered Saline (PBS) (Gibco, Life Technologies, UK) (pH = 7.2 g) and refrigerated (4 °C) overnight. The plates were washed three times with wash buffer (PBS supplemented with 0.05% Tween 20) and blocked for 2 h with 5% (w/v) of skimmed milk in PBS at room temperature. After blocking, the plates were washed three times, and diluted samples were added to wells in duplicate and incubated for an hour at room temperature. For CyRPA, MSRP5, RAMA, and SERA9, samples were diluted at 1: 100 and 1:500 for AMA-1, GLURP-RO and MSP3. After incubation, plates were washed and incubated with horseradish peroxidase (HRP)—conjugated goat anti-human IgG—(Invitrogen, USA) and detected with 3, 3’, 5, 5’- tetramethylbenzidine (TMB) (Abcam Inc., Cambridge, UK) as a substrate and absorbance read at 450 nm. Hyperimmune sera from adults living in a malaria-endemic area were used as positive control sera. Negative control sera were obtained from non-exposed anonymous Danish blood donors and were used to establish the negative cut-off.

Antibody levels were converted into arbitrary units (AU) using the formula;

$${\text{Ab}}\;{\text{levels}}/{\text{AU}} = \left( {{\text{OD}}_{{{\text{sample}}}} {-}{\text{ OD}}_{{{\text{blank}}}} } \right)/\left( {{\text{OD}}_{{{\text{positive}}\;{\text{control}}}} {-}{\text{OD}}_{{{\text{blank}}}} } \right).$$

The cut-off for seropositivity was defined as:

$${\text{Cut - off}} = \left( {{\text{Mean}} + 2 \times {\text{Standard}}\;{\text{Deviation}}} \right)\;{\text{of}}\;{\text{non-exposed}}\;{\text{individuals}}.$$

### Measurement of relative avidity

In the present study, we determined the avidities of antibodies against four selected antigens; CyRPA, MSRP5, RAMA, and SERA9. To measure the relative avidity indices, the above-described ELISA assay used to determine total IgG was modified to incorporate an additional antibody dissociation step before the HRP-conjugated secondary antibody step. In summary, diluted samples were added to each plate in quadruplicates and incubated for an hour. After washing plates, 2.4 M chaotropic agent—Sodium thiocyanate (NaSCN) (Guangdong Guanghua Sci. Tech. Co. Ltd.) was added to duplicate wells for 10 min and washed. Next, a secondary anti-human IgG antibody was added, and plates developed as stated above. Relative avidity was defined as the fraction of antibodies that remained bound to the antigen after treatment with the chaotropic agent.

$${\text{Therefore}},\;{\text{the}}\;{\text{Avidity}}\;{\text{index}} = \left( {\left[ {{\text{OD}}\;{\text{with}}\;{\text{NaSCN}}\;{\text{treatment}}/{\text{OD}}\;{\text{without}}\;{\text{NaSCN}}\;{\text{treatment}}} \right] \times 100} \right).$$

To overcome the limitation of poor resolution as a result of low ODs, only samples that were seropositive to a particular antigen were included in the assessment of the avidity index (AI).

### B cell phenotyping

PBMCS were thawed in R10 (RPMI 1640 supplemented with L-glutamine, Penicillin/Streptomycin, and 10% FBS), washed, and rested in an incubator (37 °C) for an hour. Following cell counting, 1 × 10^6^ cells were stained with Alexa Flour 700 Fixable Viability stain (FVS) (BD Biosciences) for 15 min at 4 °C. Subsequently, the cells were washed and stained at 4 °C for 30 min with a cocktail of fluorophore-conjugated antibodies in a staining buffer (PBS supplemented with 0.09% Sodium azide and 2% FCS). The fluorophore conjugated antibodies used were CD10PE (clone HI10a), CD19 BV786 (clone SJ25-C1), CD20BV737 (clone 2H7), CD23 Alexa 700 (clone M-L233), CD27 BV421 (clone M-T271), CD38 APC (clone HIT2), IgD PECy7 (clone IA6-2), IgG FITC (clone G18-145) and IgM BV605 (clone G20-127) (all from BD Biosciences). The stained cells were processed on LCS Fortessa X-20 flow cytometer (BD Biosciences) and data was analyzed using the FlowJo software Version 10.7.2.

To characterize the different B cell compartments, the following markers were used; classical memory B cells (CD19 + CD10- IgD- CD27 + CD21^high^), atypical memory B cells (CD19 + CD10-, IgD- CD21- CD27-), plasmablast/antibody-secreting cells (CD10- IgD- CD19^low^, CD20^low^ CD27^high^ CD38^high^), tissue-like memory B cells (CD 27- CD21-), Naive B cells (CD19 + , IgM + , IgD +), activated B cells (CD19 + CD20 + CD25 + CD30 +).

### In vitro growth inhibition

Plasma from individuals was tested for its ability to inhibit *P. falciparum* 3D7 parasite strain in vitro as described previously^41^ with the modification of using plasma in place of total purified IgG fractions Assays were set up with mature late-stage parasites with parasitemia between 0.5% and 0.8%. Plasma was heat-inactivated at 56 °C for 20 min before testing. Parasites were added to 96-well plates at 1% hematocrit in the presence of immune plasma diluted at 1:30 and tested in triplicates. The plates were incubated at 37 °C in a gas mixture of 2% O2, 5.5% CO2, and 92.5% N2 for 40 h. Parasite growth was estimated biochemically using lactate dehydrogenase assay. Plates were read at an absorbance of 460 nm. Percentage inhibition was calculated as; 100 − [(OD650 of infected RBCs with tested IgG − OD650 of normal RBCs only)/(OD650 of infected RBCs without any IgG − OD650 of normal RBCs only) × 100].

### Total IgG binding of merozoites by flow cytometry

Free merozoites were isolated from schizonts by passing 3D7 *P. falciparum* culture through a 1.2 µm filter. The filtrate was centrifuged at 2100×*g* for 10 min to harvest merozoites. The pellet was washed twice and resuspended in 0.5% bovine serum albumin (BSA) + phosphate buffered saline (PBS) (wash buffer). Merozoites were plated in a 96-well round bottom plate and incubated with plasma samples from immune adults at a dilution of 1:50 for 1 h at room temperature. Plasma from Danish individuals were used as non-exposed controls. After incubation, plates were centrifuged at 2100×*g* for 5 min at room temperature and washed twice in wash buffer. Subsequently, the merozoites were stained with goat anti-human polyclonal IgG PE-Fc antibody (1:200) in PBS for 20 min on ice. After staining, the merozoites were washed twice and fixed with 1% paraformaldehyde (PFA) in Hoechst live/dead staining for 30 min. The merozoites are washed and resuspended in wash buffer and 2500 events were recorded by flow cytometry. To analyse, merozoites were first gated based on forward scatter and side scatter. Secondly, a gate was set on singlets and then live/dead staining. Finally, a gate was set to profile merozoites in the forward scatter/PE frame. Mean fluorescence intensities (MFI) were estimated using FlowJo™ v10.8 Software (BD Life Sciences).

### Statistical analyses

Statistical analyses were performed using Graph Pad Prism Software Version 9.0. and R software. The non-parametric Mann–Whitney U test was used to analyze the differences in the antibody titers, antibody avidities, and the different B cell phenotypes in the high and low transmission intensity regions. Antibody breadth score was estimated by catergorizing antibody levels based on quartiles^50^. Antibody levels were designated 0,1,2,3 for lowest, second, third and highest quartile respectively. Spearman rank’s correlation was used to test correlations between variables. Principal component analysis was conducted using factoextra and corrplot packages in R. All comparisons were two-tailed and *P*-value < 0.05 was considered statistically significant.

## Data Availability

All data generated or analysed during this study are included in this published article (and its Supplementary Information files).
